# Increasing evidence of low lymphatic filariasis prevalence in high risk *Loa loa* areas in Central and West Africa: a literature review

**DOI:** 10.1186/s13071-018-2900-y

**Published:** 2018-06-15

**Authors:** Louise A. Kelly-Hope, Janet Hemingway, Mark J. Taylor, David H. Molyneux

**Affiliations:** Liverpool School of Tropical Medicine, Liverpool, L3 5QA UK

**Keywords:** *Loa loa*, Loiasis, Tropical eye worm, *Chrysops*, Vector control, Lymphatic filariasis, LF, Onchocerciasis, Neglected tropical diseases, NTDs, Africa, IVM, Integrated vector management

## Abstract

In West and Central Africa, there is a need to establish the prevalence of *Wuchereria bancrofti* in areas that are co-endemic for *Loa loa,* in order to implement the appropriate strategies to scale-up interventions for the elimination of lymphatic filariasis (LF). Due to the risk of severe adverse events (SAEs) to ivermectin in individuals with high *L. loa* microfilaraemia, the current strategy recommended by the World Health Organization (WHO) is twice yearly mass drug administration (MDA) with albendazole, supplemented by vector control targeting the *Anopheles* vectors. Defining *W. bancrofti* prevalence in areas co-endemic with *L. loa* is complicated by the cross-reactivity of rapid diagnostic immunochromatographic card tests (ICT), widely used for LF mapping, in individuals with high *L. loa* microfilaraemia. This has probably resulted in the overestimation of LF prevalence, triggering the implementation of MDA strategies, which may be unnecessary and wasteful of the limited resources for elimination programme implementation. Here we review the literature and present historical evidence, which uniformly highlight low or no prevalence of *W*. *bancrofti* infection and/or clinical LF cases across five Central African countries, in more than 30 different geographical areas covering 280 individual sites and > 22,000 individuals tested within high risk *L. loa* areas. This highlights the very limited information available on LF prevalence in *L. loa* areas, and potentially has major policy implications, which could shift the focus towards revised mapping criteria to verify low or no *W*. *bancrofti* prevalence in high risk *L. loa* areas. In this situation, revising the current WHO strategy from MDA, to focus more on ensuring high and effective vector control, through insecticide treated/long-lasting impregnated bednets (ITNs/LLINs), integration of point-of-care test-and-treat options into health systems, and consolidating closer links with the malaria control programme may be a more effective and appropriate use of the limited resources and drug donations available for LF elimination.

## Background

Lymphatic filariasis (LF) is a disabling parasitic disease transmitted by mosquitoes [1]. It is targeted for elimination and is one of the five neglected tropical diseases (NTDs) that are primarily controlled by preventive chemotherapy. The Global Programme to Eliminate LF (GPELF) has worked towards elimination for nearly two decades; first by interrupting transmission with mass drug administration (MDA) using different regimen combinations of albendazole, ivermectin and diethylcarbamazine (DEC), and second by alleviating suffering through morbidity management and disability prevention (MMDP) [2, 3]. Overall significant progress has been made towards elimination, but greater efforts are required in many countries in the World Health Organisation (WHO) African Region, where many countries remain behind the elimination targets [4].

In 2016, the African region was estimated to have 371.2 million people requiring MDA across 32 endemic countries, with a reported coverage of 56.9% [3]. While this marks an increase in coverage from previous years, there are still 17 African countries yet to start or scale-up MDA to full nationwide geographical coverage. The reasons for limited coverage are complex [5] and related to several factors including - but not limited to - political will, conflict, financial commitment, technical support, difficult to access populations, stakeholder interest, poverty, poor infrastructure, limited human capacity, competing priority diseases, and co-endemicity with the filarial parasite *Loa loa*, which causes the disease known as Tropical eye worm or loiasis.

**Fig. 1:**
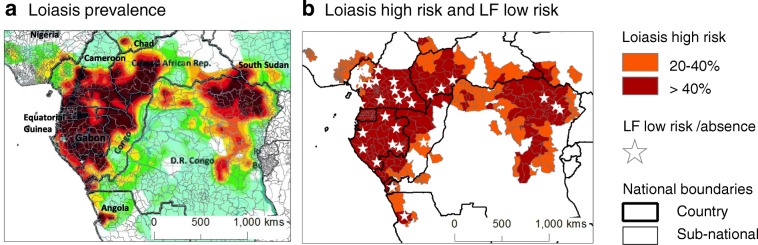
Maps of loiasis prevalence and study areas with low LF risk. **a** Loiasis prevalence. **b** Loiasis high risk and LF low risk

*Loa loa* occurs in 10 countries in Central and West Africa (Fig. 1a) [6] and poses two problems for LF elimination. First, the use of ivermectin presents a risk of severe adverse events (SAEs) to individuals with high *L. loa* parasitaemias, i.e. > 30,000 microfilariae (Mf) per millilitre blood, and can induce encephalopathy, coma and death [7, 8]; secondly by complicating the mapping of LF distribution as the standard rapid antigen diagnostic tests for LF [e.g. BinaxNOW Filariasis immunochromatographic test (ICT) and/or the new Alere Filariasis Test Strip (FTS Alere, Scarborough, ME, USA)] [9], cross-react with *L. loa,* resulting in false positives for *Wuchereria bancrofti*, with strong associations between ICT positivity and *L. loa* Mfs [10–13], e.g. around 30% and 50% of ICTs reacting to *L. loa* when densities were > 15,00 Mf/ml and > 30,000 Mf/ml respectively [13].

The WHO recommends an alternative strategy for LF elimination in *L. loa* co-endemic areas, including twice yearly MDA with albendazole supplemented with vector control, primarily insecticide-treated/long-lasting bednets (ITNs/LLINs), which control *Anopheles* mosquitoes that transmit both LF and malaria [14]. In addition, a basic five step model for national LF programmes to work at implementation unit level has been developed to supplement the WHO guidelines and help initiate treatment plans [15]; however, countries are still struggling to implement the strategy. This is complicated by the lack of clarity on the extent of LF prevalence and evidence that *W. bancrofti* transmission is absent or low in countries with high risk of *L. loa*, such as Cameroon and Gabon [3, 16, 17]. This raises questions on how to accurately map LF in high risk *L. loa* areas and whether LF MDA in these areas is warranted.

Here we review existing literature on *W. bancrofti* prevalence in high risk *L. loa* areas, to highlight the extent of *W. bancrofti*-*L. loa* co-endemicity. This may further help to refine the needs of national programmes, and current WHO strategies [14, 15].

## Geographical area and search strategy

High risk *L. loa* areas were defined as those with > 40% loiasis prevalence determined by the Rapid Assessment Procedure for Loiasis (RAPLOA) [18, 19], which correlates to 20% Mf prevalence and high risk of SAEs (Fig. 1a). The geographical high risk *L. loa* areas and the risk of LF prevalence mapped within these parameters are highlighted in Fig. 1b.

A literature search and collation of data was conducted using PubMed, JSTOR, SCOPUS and Google online sources. Search terms, and combinations thereof, included *Wuchereria bancrofti*, lymphatic filariasis, elephantiasis, *Loa loa*, loiasis, and tropical eye worm. Further sources of information were found within reference section of documents. Due to the cross-reactively problem associated with antigen tests, studies only using these tests to determine LF endemicity were excluded.

## Evidence from the existing literature

In total, 16 documents with information on LF prevalence in humans in high risk *L. loa* areas were found between 1928 to 2018. The documents ranged from historical review articles with anecdotal reports, conference abstracts to specific research articles. Most documents with specific data were from Cameroon, Gabon and DRC, and are summarised below and in Table 1.

**Table 1:** Summary of studies examining *Wuchereria bancrofti* human infection

Country	Study year^a^	Province/District	Diagnostic	Number of sites	Number sampled	Reference
Angola	2015	Bengo Province	Clinical	29	2017	[20]
DRC	2013	Oriental Province	Mf	30	2724	[10]
	1974^a^	Mayombe, Bas Congo Province	Mf	32	2476	[22]
Cameroon	2018	East, Central, South, Littoral	Clinical; Mf; Wb123; qPCR	29	4698 (24)^c^	[23]
	2017^b^	South, Central, South-east, North West, Far North	Wb123; qPCR	50	5000	[16]
	2016	East, Central, South, Littoral	Mf	31	14,577 (185)^c^	[17]
	2016	Messok District, Haut-Nyong Department	qPCR	8	1085	[24]
	2013	Lolodorf surrounds	Wb123; qPCR	26	1812	[13]
	2013	East, North-west, South-west	Mf; Clinical	42	2190 (24)^c^	[11]
	1990^a^	Goura, Badissa Nyamanga	Mf	3	1324	[25, 29]
	1975	Department Mifi	Mf; Clinical	6	1004	[25, 28]
	1957^a^	Yoko Region	Mf	1	50	[25, 27, 36]
Total				287	38,957 (22,399)^d^	

^a^Indicates publication year, when study year is not stated

^b^The exact numbers in the high risk *Loa* areas is unclear until publication i.e. the North, West, far West have minimal or no overlap with high risk areas, and therefore related numbers are likely not to count

^c^Indicates the number of individuals initially examined for clinical conditions or using ICT or FTS (with positive cases examined further using different diagnostic tests)

^d^Indicates total number of individuals examined (total number excluding ICT or FTS)

### Angola

In 2014, a micro-mapping clinical survey was conducted in Bengo Province in the northern region of Angola including 2017 individuals from 29 communities. Clinical cases of LF were confirmed by medical officers. Eight individuals had limb lymphoedema (0.4%) and 20 men had hydrocoele (2.6%) [20]. While clinical cases do not necessarily represent recent transmission, nor define endemicity, the relatively low numbers are in line with historical surveys in the same region in the 1960s [21], and highlight the probable distribution of isolated foci of *W. bancrofti* in northern Angola, with endemic zones in Cabinda District and the northern region of Zaire District.

### Democratic Republic of the Congo

In 2011–2013, a seroprevalence survey was conducted in 30 villages in the Oriental Province in eastern DRC including 2724 individuals (aged ≥ 14 years) from the Ituri region (Mambasa Territory) and Haute Uele region (Watsa Territory) [10]. The presence of *W. bancrofti* was assessed by examining night blood slides for Mf by microscopy and parasite DNA by quantitative real-time polymerase chain reaction (qPCR). Only one individual was positive for *W. bancrofti* DNA.

In 1974, a study in 32 villages in the Mayumbe region on the western coast of DRC, close to the mouth of the Congo River, including 2476 adult individuals who had lived in that region for at least 5 years. Only one village was positive for *W. bancrofti* by night blood slide Mf microscopy [22].

### Cameroon

In 2017, two studies from Cameroon highlighted the absence of *W. bancrofti* in *L. loa* endemic areas [16, 17]. Wanji et al. [16] reported a seroprevalence study of 5000 individuals from 50 villages across the South, Central, South-east, North-west, Far-north areas of the country, which detected no *W. bancrofti* positivity using two different antibody Wb123 tests, examining night blood slides for Mf and confirming selected samples with pPCR methods. Similarly, Biholong et al. [17] reported on a seroprevalence study conducted in 2010–2012 of 14,577 individuals (aged ≥ 9 years), across 31 health districts which detected no *W. bancrofti* Mf by night blood slide in 185 of the 235 individuals who were initially found to be antigen-positive using FTS.

In 2016, a study examining the geographical distribution of podoconiosis using parasitological, serological and clinical evidence to exclude the causes of lymphoedema, including LF was conducted across ten Regions of Cameroon. In the four high risk *L. loa* regions, i.e. the Central, East, Littoral and South Regions, a total of 4698 people from 29 communities were examined. In total 24 lymphoedema cases were found in these four Regions, and of these one was FTS-positive (Littoral Region) and none were positive by Mf, Wb123 and qPCR test [23].

Additional findings in 2013 and 2016 suggested no LF. These included a seroprevalence study in eight villages in Messok District [24], including 1085 individuals (the majority aged > 15 years) in 2016, and a seroprevalence study in 26 communities within 50 km of Lolodorf, southern Cameroon, including 1812 individuals (aged ≥ 5 years) in 2013 [13]. The absence of *W. bancrofti* was confirmed by qPCR and Wb123 assays in 52 individuals initially found to be antigen-positive using ICTs. All other ICTs were negative.

Another seroprevalence study in 42 communities in the East, North-west and South-west of the Cameroon rainforest belt, including 2190 individuals (aged > 10 years) in 2013, found no *W. bancroft*i Mf by night blood slide in 24 individuals that were antigen-positive using ICTs. Twenty cases of lymphoedema were found and none of them were ICT-positive. No cases of hydrocele were found.

A review by Boussinesq in 1999 [25], of historical studies published in French, highlighted that in 1928, Sharp [26] found very low *W. bancrofti* Mf prevalence 0.4–2.0% in Mamfe region; in 1957, Languillion [27] reported no *W. bancrofti* Mf in 50 individuals from Yoko region; in 1975, Brengues [28] reported no *W. bancrofti* filaria in six localities in the Department of Mifi including 1004 individuals, and 32 cases of elephantiasis among 2395 individuals clinically examined; in 1990, Lochouarn [29] found no *W. bancrofti* Mf in three villages, Goura (Department du Mbam et Kim), Badissa and Nyamanga (Mbam et Inoubou) including 1324 individuals (aged > 10 years).

### Excerpts from historical and/or anecdotal reports

While rigorous data on LF distribution in *L. loa* areas is limited, there are many anecdotal and historical reports that *W. bancrofti* across Central Africa is low or absent. These are presented here as excerpts with the approximate decade and country of the review, study or publication to provide perspective on time frame and place.

- ✓ *“W. bancrofti is said not to be found from the Uele river in the Belgian Congo to the Coast or from the Ouham river to Gabon. Nor has it been found in Gabon, according to Galliard. At M’Bomou (a head tributary of the Ubangui river), W. bancrofti was not found in 400 night bloods, although elephantiasis occurs; this is said to be due to Onchocerca.*” (~1910–1950s; Gabon) [30–32]
- ✓ *“… elephantiasis, on the contrary, is rather rare and a very limited distribution*” (~1910s; French Equatorial Africa) [32]
- ✓ *“In the Cameroons, we have never found the microfilariae in night surveys in five large villages in the rainforest, not during the day in many thousands of bloods films. Elephantiasis only occurs in the rainforest where infection with O. volvulus is found.*” (1950s; Cameroon) [30]
- ✓ *“It has never been encountered in many thousands of similar blood films taken in the Cameroons.*” (~1950s; Cameroon) [33]
- ✓ *“In most of Belgian Congo, however, filariasis seems to be rare and unimportant.*” (~1950s; Belgian Congo) [30]
- ✓ *“*… *in 1924*, *an extensive investigation into the incidence of infection by L. loa and A. perstans, and found that the percentage of population infection with L. loa was 17, with A. perstans 77, and with F. bancrofti only 0.4.*” (~1920s; Cameroon) [26, 30]
- ✓ *“In Ayos, W. bancrofti is never seen.*” (~1930s; French Cameroons) [30]
- ✓ *“In the Bankim district, located in the Tikar plain, the 10 surveyed villages were all hyperendemic for loiasis. … The low prevalence of hydroceles suggests that lymphatic filariasis is not endemic in this study area.*” (~2000s; Cameroon) [34]
- ✓ *“According to Languillon (1957) the microfilaria rate is 16% in the savanna region of the north (Diamare and Benoue), but falls to 0.8% in the forest zone of the south…*” (1950s; Cameroon) [27, 35]
- ✓ *“Infection with W. bancrofti is considered to be slight but there is little evidence on the subject (Hamon et al. 1967). Brumpt et al. (1972) who examined 100 persons in two small villages in the north near the Chad frontier, detected one case of infection with this parasite.*” (1960s; Central African Republic) [35–37]
- ✓ *“ … In the western region of Nigeria, with special reference to the Ibadan area, W. bancrofti was very rare in this region. … On the other hand, Ibadan lies in the heart of the Loa belt of West Africa and its infection there is common*.” (~1970s; Nigeria) [38]
- ✓ *“For the Gabonese population one can approximately say that 3 out of 5 adult patients have filariasis, two being D. perstans and one L. loa. Consequently, these affections represent a major public health problem. Finally, one must note that no cases of lymphatic filariasis has been identified.*” (~ 1980s; Gabon) [39]
- ✓ *“Because only a few infections were identified across Gabon … undergoing remapping to determine whether MDA for LF is warranted,*” (~2010s; Gabon) [3]

## Conclusions

This review highlights the very limited information available on LF prevalence in *L. loa* areas. The data that are available are consistent with anecdotal historical reports indicating that LF is very low or absent in high risk *L. loa* areas. The distinct ecological niches of the filarial parasites and their various vectors may explain these differences, i.e. *Anopheles* species transmitting *W. bancrofti* may prefer savanna-like habitats more than the deep forested ecology, where the *L. loa* vector *Chrysops* species thrive, and there may be competitive exclusion, given the co-endemicity of five human filarial parasites in these countries [40]. Regardless, these findings have major implications for co-endemic countries, and GPELF, and may significantly reduce the need for MDA for millions of people across vast geographical areas of Central and West Africa as noted recently in Cameroon [16, 17], with consequent resource implications. It is important that further confirmatory mapping is conducted, with appropriate population-based sampling strategies, including individuals over five years of age. Sentinel site surveys are not sufficient as the geographical scale is not sufficiently refined to detect focal transmission hotspots in low endemic areas. Targeted micro-mapping may be more suitable [20, 41, 42], especially in areas that are ecologically diverse where the co-endemicity may vary and different diagnostic tools may be required. Better delineation of where the rapid antigen test for *W. bancrofti* can or should be used is important (e.g. in low risk *L. loa* areas) as they may help to identify regions that do not require MDA [13]. Equally, it will be important to identify areas where alternative *W. bancrofti* diagnostics may be more appropriate to use such as the antibody Wb123-based mono or biplex test, qPCR or microscopy (e.g. high risk *L. loa* areas). Further, it will also be important to distinguish between LF clinical manifestations and other diseases with similar clinical presentations, notably podoconiosis (or non-filarial lymphoedema), which appears to be endemic in some of these countries [23, 43]. Monitoring the impact of other interventions used in these areas will be critical. The widespread distribution of community-directed treatment with ivermectin (CDTI) for onchocerciasis control may have impacted *W. bancrofti* transmission as shown elsewhere [44, 45]. Similarly, the recent significant scale-up of vector control for malaria, primarily ITNs/LLINs, which has reduced malaria prevalence [46], may have reduced *W. bancrofti* prevalence, as also demonstrated elsewhere [47, 48]. Revising the current WHO strategy for *L. loa* areas from MDA, to focus more on ensuring effective vector control, through ITNs/LLINs, integrating point-of-care test-and-treat options into health systems and consolidating closer links with the malaria control programme, may be a better use of the limited resources available for LF elimination programmes.

## Abbreviations

CDTI: Community-directed treatment with ivermectin
DEC: Diethylcarbamazine
DRC: Democratic Republic of the Congo
FTS: Filariasis test strip
GPELF: Global Programme to Eliminate Lymphatic Filariasis
ICT: Immunochromatographic test
LF: Lymphatic filariasis
MDA: Mass drug administration
Mf: Microfilaria
NTDs: Neglected tropical diseases
qPCR: Quantitative real-time polymerase chain reaction
RAPLOA: Rapid Assessment Procedure for Loiasis
SAE: Severe adverse event
WHO: World Health Organization
